# The Long Noncoding RNA MEG3 Is Downregulated and Inversely Associated with VEGF Levels in Osteoarthritis

**DOI:** 10.1155/2015/356893

**Published:** 2015-05-21

**Authors:** Wei Su, Wen Xie, Qingkun Shang, Bing Su

**Affiliations:** ^1^Department of Orthopedics, The Third Affiliated Hospital, Xinxiang Medical University, Xinxiang, Henan 453003, China; ^2^Luoyang Orthopedic Hospital, Luoyang, Henan 471002, China; ^3^Department of Biostatistics, School of Public Health and Health Professions, The State University of New York, Buffalo, NY 14214, USA; ^4^Biomedical Research Institute, Shenzhen PKU-HKUST Medical Center, Shenzhen, Guangdong 518036, China

## Abstract

Osteoarthritis (OA) is becoming a major public health problem in China, especially considering the increase in average life expectancy of the population. Thus, enhanced understanding of the molecular changes associated with OA is urgently needed to develop more effective strategies for the diagnosis and treatment of this debilitating disease. LncRNAs play an important role in the processes of bone and cartilage development. Maternally expressed gene 3 (MEG3) is a maternally expressed lncRNA and may function as a tumor suppressor by inhibiting angiogenesis. OA is closely associated with angiogenesis and the inhibition of angiogenesis presents a novel therapeutic approach to reduce inflammation and pain in OA. In this study, we detected the mRNA expression of MEG3 and VEGF in articular cartilage samples from 20 OA patients and 10 healthy volunteers by real-time RT-PCR. VEGF protein is detected by ELISA in cartilage samples. The results show that human MEG3 is significantly downregulated in OA patients compared to normal cartilage samples. However, higher levels of VEGF mRNA and protein are found in OA compared to the control. Moreover, MEG3 levels are inversely associated with VEGF levels, suggesting that MEG3 may be involved in OA development through the regulation of angiogenesis.

## 1. Introduction

Half of the population, aged 65 and older, suffer from osteoarthritis (OA) worldwide [1]. OA is also becoming a major public health problem in China considering the increasing life expectancy of the population. To date, the causes of OA remain unclear and a definitive cure is still not available. Therefore, the discovery of new biomarkers would facilitate the development of tailored preventive and therapeutic approaches and minimize the expected negative impacts of the disease. OA is closely associated with inflammation and angiogenesis. Targeting synovial membrane angiogenesis may present an important strategy to OA treatment and reduction of disease symptoms [2].

Noncoding RNAs (ncRNAs) belong to a newly identified class of RNA molecules which do not have protein-coding capacity [3]. According to their size, ncRNAs are divided into two main categories: small ncRNAs, such as microRNAs (miRNAs), and long noncoding RNAs (lncRNAs) [4, 5]. Profiling studies suggest that miRNAs are aberrantly expressed in OA, which may have implications in the regulation of cartilage integrity through inflammation, cellular communication, or cell death [6].

On the other hand, increasing data indicate that lncRNAs play an important role in the processes involved in bone and cartilage development [7]. Aberrant expression profile of lncRNAs in OA has been reported recently, suggesting their potential contributions in OA progression and a potential for their use as biomarkers for disease diagnosis and treatment [8].

Maternally expressed gene 3 (MEG3) is a maternally expressed lncRNA and a tumor suppressor gene located in chromosome 14q32 [9]. MEG3 may inhibit tumor progression by inhibiting angiogenesis [10]. In the present study, we examined the expression of human MEG3 in cartilage samples from OA patients and healthy subjects. We also investigated the potential relationship of MEG3 expression and VEGF level in OA.

## 2. Materials and Methods

### 2.1. Specimens

Osteoarthritic knee cartilage samples from 20 OA patients, aged 50–65 years, were obtained from the Third Affiliated Hospital of Xinxiang Medical College. Normal cartilage samples with no gross sign of degradation were collected from 10 control subjects with no history of OA or rheumatoid arthritis. Written consents were obtained from all subjects prior to the recruitment and the study protocol was approved by the Ethics Committee of the Third Affiliated Hospital of Xinxiang Medical College. The clinical characteristics of the subjects are listed in Table 1.

### 2.2. RNA Extraction and Quantitative Reverse Transcriptase PCR (qRT-PCR)

Total RNA was isolated from cartilage tissues using the RNeasy kit (Qiagen, Grand Island, NY) according to the manufacturer's instructions. Reverse transcription reactions were carried out with 1 *μ*g total RNA using the PrimeScript RT reagent kit (TaKaRa BIO, Shiga, Japan). Random hexamer primers were used in the RT reactions. Real-time PCR was performed on a Bio-Rad CFX-96 real-time PCR system using SYBR Premix DimerEraser kit (TaKaRa, Shiga, Japan) following the manufacturer's instructions. MEG3 and the primer sequences of three VEGF subgroups were obtained from previous publication [11, 12]. GAPDH was used as a housekeeper gene for the qRT-PCR reactions. Each test was done in triple replication and the 2^−ΔCt^ method was used to calculate the expression of lncRNA MEG3 in tissue samples. The sequences of the PCR primers used are listed in Table 2.

### 2.3. Quantification of Vascular Endothelial Cell Growth Factor (VEGF) by ELISA

VEGF detection by ELISA was conducted as previously reported [13]. Briefly, 100 mg fresh weight of cartilage tissue was crushed under liquid nitrogen and homogenized in 150 mM NaCl and 20 mM Tris HCl buffer, pH 7.4. A soluble fraction was obtained by centrifugation at 48,000 g for 60 minutes, and 100 mL aliquots were analyzed by a sandwich ELISA (R&D Systems, Minneapolis, MN) that detects all VEGF splice forms. The amount of VEGF was expressed as ng/g dry tissue.

### 2.4. Statistical Analysis

The results were presented as mean ± SD. SAS 9.3 software (Site 70135983. SUNY UB, USA) was used for all statistical analyses. A *P* value of <0.05 was considered significant.

## 3. Results

### 3.1. Clinical Characteristics of Study Subjects

Twenty OA patients and 10 healthy age-matched individuals were recruited to this study. When recruited, the average ages of normal individuals and OA patients were 58.9 ± 6.7 and 59.4 ± 6.2 years, respectively. No significant difference in age and sex distribution was found among the two study groups (*P* > 0.05) (Table 1).

### 3.2. MEG3 Expression Was Significantly Downregulated in OA Patients

**Figure 1:**
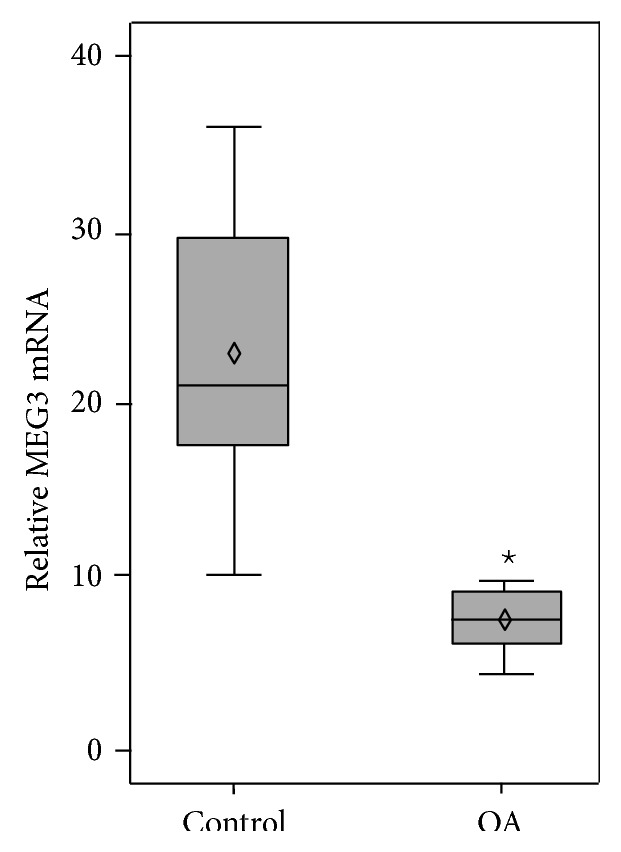
Downregulation of lncRNA MEG3 expression in osteoarthritis (OA) cartilage samples. qRT-PCR analysis of MEG3 expression in cartilage samples from 10 healthy volunteers and 20 OA patients. GAPDH was the internal control (^⋆^ *P* < 0.01).

To study the mRNA expression level of MEG3 in the cartilage samples of OA patients, qRT-PCR was performed in samples from 20 OA patients and 10 normal individuals. The expression level of MEG3 was significantly lower in samples of the OA group compared with the normal group (*P* < 0.01) (Figure 1).

### 3.3. Upregulation of VEGF mRNA and Protein in OA Patients

**Figure 2:**
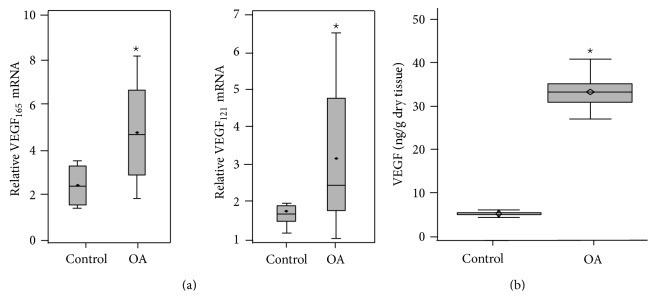
Upregulation of VEGF mRNA and protein in OA cartilage samples. (a) qRT-PCR analysis was performed to examine MEG3 RNA levels in cartilage tissues from 20 OA patients and 10 controls. The results shown are from three independent qRT-PCR runs and are reported as the mean ± SD. ^⋆^ *P* < 0.01. (b) The cartilage tissues from 20 OA patients and 10 controls were homogenized in buffer, and concentrations of immunoreactive VEGF were determined in homogenates by ELISA that detects all VEGF splice variants. The results shown are from three repetitions and are reported as the mean ± SD. ^⋆^ *P* < 0.01.

Angiogenesis contributes to the OA synovial inflammation and is associated with disease severity. MEG3 has been reported to inhibit angiogenesis in cancer [10]. Therefore, we next investigated the mRNA and protein expression of VEGF, a key factor in angiogenesis, in OA cartilage tissues. The qRT-PCR results show that mRNA expression of VEGF_121_ and VEGF_165_ was significantly higher in OA samples than in control samples (*P* < 0.01) (Figure 2(a)). This result is in line with previous reports that VEGF_121_ and VEGF_165_ are two major isoforms and play a major biological role in the VEGF family [14]. We further compared the expression of VEGF protein in two groups by ELISA. VEGF protein levels were significantly higher in OA samples than in normal samples (*P* < 0.01) (Figure 2(b)).

### 3.4. The Correlation of MEG3 with VEGF

**Figure 3:**
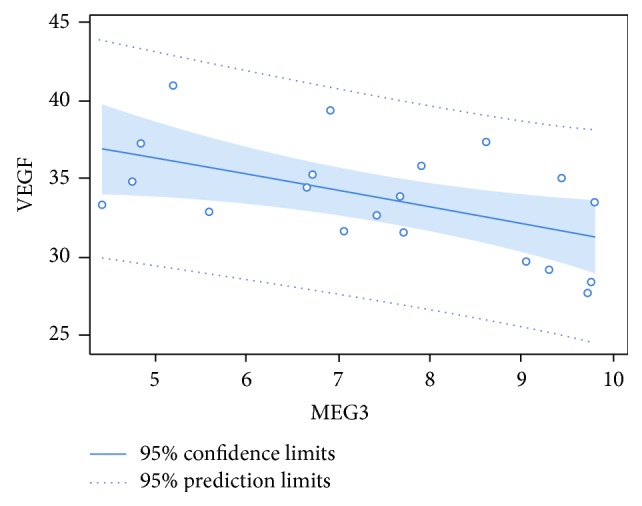
The correlation of MEG3 with VEGF. Pearson correlation coefficient was calculated in SAS 9.3 as a measure of the degree of linear dependence between VEGF and MEG3 in OA group.

To study whether MEG3 levels are associated with VEGF expression, Pearson correlation coefficient was calculated as a measure of the degree of linear dependence between VEGF and MEG3 in the OA group. As seen in Figure 3, Pearson's *r* = −0.53409 with *P* = 0.0153, which indicates there is significant negative correlation between VEGF and MEG3 given a significance level of *α* = 0.05.

## 4. Discussion

Despite its high prevalence and substantial public health impact, OA etiology is not fully understood. The identification of genes and noncoding RNAs associated with OA will help reveal the underlying molecular mechanisms and lead to the development of targeted therapies.

At present, 27 transcripts of MEG3 have been reported (http://www.ensembl.org/). Zhang et al. identified several MEG3 cDNA isoforms by screening a human fetal liver cDNA library and found that all MEG3 isoforms have the same ability to stimulate p53-mediated transactivation and suppress tumor cell growth, suggesting that MEG3 isoforms have similar functions [15]. Hence, we evaluated the expression levels of MEG3 in OA cartilage tissues using the primers against all MEG3 splicing variants. We demonstrated that MEG3 expression was significantly decreased in OA patients compared with the normal control (*P* < 0.01).

Gordon et al. [10] discovered increased expression of proangiogenic genes in the brains of mouse MEG3-null embryos, suggesting that deletion of MEG3 promotes angiogenesis. Many studies emphasize the importance of angiogenesis in OA and its contribution to progressive joint damage [16]. VEGF has been shown to regulate hypertrophic cartilage remodeling, ossification, and vascular invasion of growth plate cartilage [17]. The invasion of blood vessels into cartilage, which is normally avascular, is the crucial first step in ossifications, and the vasculature provides a conduit for the recruitment of the cell types involved in cartilage resorption and bone deposition [18].

To determine whether there is a relationship between MEG3 and VEGF in OA, we measured the VEGF levels by qRT-PCR and ELISA. Compared to the normal control, the expression of VEGF isoforms is significantly increased in OA samples, especially the secreted VEGF_121_ and VEGF_165_. Further, ELISA results indicate that the level of VEGF protein also increased in OA cartilage tissues. Association analysis reveals that MEG3 expression is inversely correlated with VEGF expression in OA, suggesting that enhanced angiogenesis may be a mechanism by which inactivation of MEG3 contributes to OA development.

The detailed mechanisms by which MEG3 inhibits angiogenesis remain to be determined. It has been shown that MEG3 stimulates p53-mediated transcriptional activation [19, 20]. P53 is known to negatively regulate VEGFA transcription through binding to the transcription factor Sp1 sites on the VEGFA promoter [21]. Therefore, it is possible that the loss of MEG3 leads to reduced p53 activity and increased transcription of VEGFA. These results suggest that MEG3-mediated inhibition of angiogenesis and VEGF may be through p53 pathways.

In conclusion, we demonstrate that MEG3 is significantly downregulated in human OA cartilage tissues compared with a normal control. These findings indicate that MEG3 may be a potential target for OA therapy.

## Figures and Tables

**Table 1:** Clinical characteristics of the study subjects.

Characteristics	Normal	OA	*P* value
Sex			
Male	6	12	
Female	4	8	>0.05
Ages (yr)			
Mean	58.9 ± 6.7	59.4 ± 6.2	
Range	47–65	53–69	>0.05

**Table 2:** PCR primers used in this study.

	Sense	Antisense
MEG3	5′-CTGCCCATCTACACCTCACG-3′	5′-CTCTCCGCCGTCTGCGCTAGGGGCT-3′

VEGF_121_	5′-CCCTGATGAGATCGAGTACATCTT-3′	5′-GCCTCGGCTTGTCACATTTT-3′
VEGF_165_		5′-AGCAAGGCCCACAGGGATTT-3′
VEGF_189_		5′-AACGCTCCAGGACTTATACCG-3′

GAPDH	5′-GAAGGTGAAGGTCGGAGTCA-3′	5′-GAAGATGGTGATGGGATTTC-3′
